# A Random Regression Model Based on a Single-Step Method for Improving the Genomic Prediction Accuracy of Residual Feed Intake in Pigs

**DOI:** 10.3389/fgene.2021.769849

**Published:** 2022-02-01

**Authors:** Ye Wang, Chenguang Diao, Huimin Kang, Wenjie Hao, Raphael Mrode, Junhai Chen, Jianfeng Liu, Lei Zhou

**Affiliations:** ^1^ National Engineering Laboratory for Animal Breeding, College of Animal Science and Technology, China Agricultural University, Beijing, China; ^2^ MARA Key Laboratory of Animal Genetics and Breeding, College of Animal Science and Technology, China Agricultural University, Beijing, China; ^3^ Guangdong Provincial Key Laboratory of Animal Molecular Design and Precise Breeding, School of Life Science and Engineering, Foshan University, Foshan, China; ^4^ Best Genetic Breeding Farm, Inner Mongolia, China; ^5^ Animal Biosciences, International Livestock Research Institute, Nairobi, Kenya; ^6^ Shenzhen Kingsino Technology Co., Ltd., Shenzhen, China

**Keywords:** residual feed intake, random regression model, animal model, genomic prediction, pigs

## Abstract

Residual feed intake (RFI) is considered as a measurement of feed efficiency, which is greatly related to the growth performance in pigs. Daily feeding records can be obtained from automatic feeders. In general, RFI is usually calculated from the total measurement records during the whole test period. This measurement cannot reflect genetic changes in different growth periods during the test. A random regression model (RRM) provides a method to model such type of longitudinal data. To improve the accuracy of genetic prediction for RFI, the RRM and regular animal models were applied in this study, and their prediction performances were compared. Both traditional pedigree-based relationship matrix (**A** matrix) and pedigree and genomic information-based relationship matrix (**H** matrix) were applied for these two models. The results showed that, the prediction accuracy of the RRM was higher than that of the animal model, increasing 24.2% with **A** matrix and 40.9% with **H** matrix. Furthermore, genomic information constantly improved the accuracy of evaluation under each evaluation model. In conclusion, longitudinal traits such as RFI can describe feed efficiency better, and the RRM with both pedigree and genetic information was superior to the animal model. These results provide a feasible method of genomic prediction using longitudinal data in animal breeding.

## Introduction

As feeding production cost is the highest among all production costs of pig farming, feed efficiency has great importance for the swine industry efficiency (Patience et al., 2015). Therefore, improving feed efficiency is vital for the whole swine industry. It not only reduces feed consumption, breeding cost, and energy consumption, but is also helpful in reducing fecal and greenhouse gas emissions (Shirali et al., 2012). In the swine industry, the ratio of feed intake to body weight gain, which is defined as feed conversion ratio (FCR), is commonly regarded as a measure of feed efficiency. However, owing to the complex association to several growth traits, direct selection for improved FCR could result in negative selection responses in back fat thickness and growth rate (Hoque et al., 2009). Thus, residual feed intake (RFI) has been proposed as another alternative measurement of feed efficiency. RFI is defined as the difference between the observed feed intake and the expected feed intake for maintenance and growth of an individual (Koch et al., 1963). Research has shown that genetic selection for RFI could improve feed efficiency and reduce feed intake without affecting growth performance (Dai et al., 2017).

RFI has aroused the interest of researchers, and many studies have shown that RFI can be defined using different methods; in particular, it can be calculated by establishing multiple linear regressions between feed intake and production performance and metabolic body weight (Cai et al., 2008; Hoque et al., 2009; Saintilan et al., 2012; Godinho et al., 2018). However, this model has some limitations. In this model, each animal has one single record for the whole test period, and it is difficult to eliminate abnormal records or reflect abnormal situations in the measurement process, such as a sudden impact of the reduction of feed intake caused by an unpredictable disease. With the development of automatic feeders and electronic identification technology (transponders), it has become increasingly convenient to collect daily feeding records accurately per feeding visit, and it also facilitates the application of random regression models (RRM) in studies on RFI. In the RRM, multiple observations of each animal for different time points are analyzed simultaneously. Bignardi et al. (2011) and Begli et al. (2018) used a longitudinal model to study the feed intake and residual feed intake traits in an F2 chickens’ population, they both found that RRM provided a good description of feeding behavior records and resulted in improved genetic gain. Shirali et al. (2014) studied residual energy intake in different growth stages of pigs, and proposed to consider the growth stage when selecting for residual energy intake because of their different genetic backgrounds. Shirali et al. (2017a), Shirali et al. (2017b) and Coyne et al. (2017) conducted longitudinal researches on genetic evaluations of feed efficiency. The potential advantages of RRM had been widely exploited in dairy cattle breeding (Schaeffer et al., 2000; Bignardi et al*.*, 2011; Kang et al., 2017). However, the study and application of RRM for RFI genetic selection are still in the early stage. There are relatively few researches comparing the accuracy of genetic evaluations from animal models and random regression models for RFI.

In this study, RRM with both traditional and pedigree-and-genomic-based relationship matrices for genetic evaluation of RFI were applied, and their predictive performance were compared with the traditional animal models.

## Materials and Methods

### Animals and Phenotypic Records

Data from a total of 1,527 Yorkshire boars with birth dates between 2017 and 2019 were collected from a breeding farm in Inner Mongolia, China. The feed intake records of these pigs were electronically measured by the automatic feeder (Nedap Pig Performance Testing equipment, https://www.nedap-livestockmanagement.com/pigfarming/solutions/performance/). Each time only one pig visits the feeder, the feeder identifies the pig’s ID and recorded the feed intake and the body weight of the pig for this visit. Back-fat thickness was measured at the end of the test period. According to the criteria proposed by Casey et al. (2005), quality control for original data and deletion of missing values were carried out. Errors in each visit were identified and counted for each day by the criteria of Casey et al. (2005), then adjusted the error-free feed intake for each pig of each day by fitting a linear mixed model with error counts and average daily gain and body weight as covariates. Finally, there were 1,440 Yorkshire boars with qualified data of the total feed intake, total weight gain, final weight, and back-fat thickness measured at the end of the test period. Total feed intake was the sum of feed intake of one pig during the whole test period, and total weight gain was the difference of the initial body weight and final body weight of the test for that pig. A total number of 1,226 boars of these 1,440 boars had longitudinal measurements of daily feed phenotypic records during the test period, such as daily gain and daily feed intake. Daily feed intake was the sum of each feed intake in a day, and daily body weight was the mode of all body weights measured in that day. Data of the first testing week for each pig were removed as this period was considered an adaptation period for pigs to adapt to the feeder.

**FIGURE 1:**
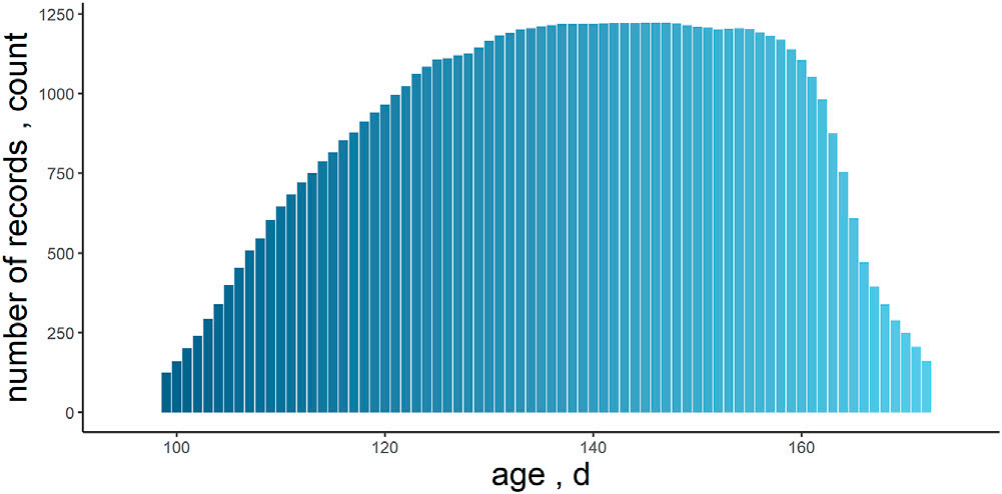
Distribution of daily records number with days of age increasing in Yorkshire.

A total number of 1,226 individuals with both kinds of phenotypes were used in the following analysis, and the descriptive statistics of their data are summarized in Table 1. The individuals recorded in the analysis were measured for at least 34 days, excluding the first week of measurements. On average, each individual had 54.01 ± 9.90 records during the testing period. For the whole test period, longitudinal feed intake phenotypes from approximately 99–172 days of age were analyzed (Figure 1).

**TABLE 1:** Mean and standard deviation of each traits.

Traits	Mean ± s.e.	Definitions
SBW, kg	49.24 ± 10.43	Initial body weight
FBW, kg	104.04 ± 11.50	Final body weight
onAGE, d	105.57 ± 10.30	Initial age of testing period
offAGE, d	165.73 ± 6.17	Final age of testing period
BFA, mm	12.15 ± 2.42	Adjusted back-fat thickness
ADG, kg/d	0.91 ± 0.13	Average daily gain

### Genotype Data

Among the 1,226 boars with phenotype, there were 900 boars which were genotyped by a self-designed single nucleotide polymorphism (SNP) chip named “CAU50K” (including 43,832 SNPs) in this study. Quality control was performed using PLINK 1.9 (Chang et al., 2015) with the following criterion: genotype call rate >95%; deviations from Hardy Weinberg equilibrium *p* > 10^−6^; minor allele frequency >0.01. Here, a total number of 35,663 SNP markers and 898 boars met the above criterion. In order to fill in missing genotypes of some individuals, complete genotypes for all the SNPs were obtained by imputation using BEAGLE 5.0 (Browning et al., 2018).

### Statistical Model and Analysis

Corresponding to different types of phenotype data, two different models were developed for the analysis of the RFI, *viz.* an animal model and random regression model (RRM).

The animal model was defined as:

$$ADFI_{ijlkm}=\mu +YS_{i}+pen_{j}+b_{1}\times ADG_{k}+b_{2}\times BFA_{k}+b_{3}\times SBW_{k}+b_{4}\times MBW_{k}+litter_{l}+a_{k}+e_{ijlkm}$$ (1)

where $ADFI_{ijlkm}$ was the average daily feed intake, μ was the overall mean, $YS_{i}$ was the fixed effect of the i th year-season, $pen_{j}\;$ was the fixed effect of the j th pen, $ADG_{k}$ , $\;BFA_{k}$ , $\;SBW_{k}$ and $MBW_{k}\;$ were the covariates of average daily gain, adjusted back-fat thickness, initial body weight and metabolic body weight for the k th individual, and metabolic body weight was average pig body weight to the power of 0.75; and these four effects had significant effects on ADFI (*p* < 0.05); $b_{1}$ , $b_{2}$ , $b_{3}$ , and $b_{4}$ were the regression coefficients of ADG , BFA , SBW , and MBW respectively; $litter_{l}$ was the l th random effect of litter; $a_{k}$ was the additive effect of RFI for the k th individual; and $e_{ijlkm}$ was the residual error.

Alternatively, the RRM was developed as:

$$DFI_{ijkt}=YS_{i}+pen_{j}+b_{1}\times ADG_{k}+b_{2}\times BFA_{k}+b_{3}\times SBW_{k}+b_{4}\times MBW_{k}+\overset{n}{\underset{m=0}{\sum }}litter_{lm}\varphi_{m}\left(t\right)+\overset{p}{\underset{m=0}{\sum }}a_{km}\varphi_{m}\left(t\right)+\overset{q}{\underset{m=0}{\sum }}p_{km}\varphi_{m}\left(t\right)+e_{ijkt}$$ (2)

where $DFI_{ijkt}$ was the daily feed intake of the k th individual, at *t*th age (days), i th year-season and j th pen, year-season ( $YS_{i}$ ) and pen ( $pen_{j}$ ) were fixed effects; $ADG_{k}$ , $BFA_{k}$ , $SBW_{k}$ , $MBW_{k}$ , $b_{1}$ , $b_{2}$ , $b_{3}$ and $b_{4}$ were the same as above; $\varphi_{m}\left(t\right)$ was the m th Legendre polynomial for animal k at age t ; $litter_{lm}$ was the m th random regression coefficient related to the l th random effect of litter; $a_{km}$ and $p_{km}$ were the m th random regressions for animal and the permanent environment effects for animal k , respectively; n was the order of polynomial for the litter effect; p and q were the order of polynomials for animal effect and permanent environmental effects for each animal, based on the Bayesian information criterion (BIC) values (Schwarz, 1978) and the complexity of the calculation, n, p and q were set as 1, 2 and 1 in this study; and $e_{ijkt}$ was the time-independent random residual error.

For the animal model and RRM, two different additive genetic relationship matrices were employed in the genetic evaluation. One was the traditional pedigree-based relationship matrix (defined as **A** matrix), and the other was pedigree-and-genomic-based relationship matrix (defined as **H** matrix) model. Therefore, four prediction models, labeled as animal model-Amat, animal model-Hmat, RRM-Amat and RRM-Hmat, were applied in this study. When the pedigree-based matrix was implemented in the above two models, the individual random additive effects of RFI a follow a normal distribution, $N\left(0,A\times \sigma_{a}^{2}\right)$ , where $\sigma_{a}^{2}$ is the additive genetic variance.

When the **H** matrix was implemented in the above two models, it is assumed that a follows $N\left(0,H\times \sigma_{a}^{2}\right)$ , where H is a combined relationship matrix constructed by both pedigree and genotype. $H^{-1}$ was computed as described by Misztal et al. (2013):

$$H^{-1}=A^{-1}+\left[\begin{array}{cc} 0 & 0\\ 0 & \tau {\left(\alpha G+\beta A_{22}\right)}^{-1}-\omega A_{22}^{-1} \end{array}\right].$$ (3)

where α , β , τ , and ω were tuned and fixed as 0.95, 0.05, 1.00 and 1.00, respectively.

Software BLUPF90 (program REMLF90 and program BLUPF90) (Misztal et al., 2002) was used to estimate the (co)variance components and perform RFI predictions with both animal model and RRM. The (co)variance components used in animal model and RRM with **H** matrix were those estimated with animal model and RRM using **A** matrix, respectively. To enable comparison of predictive ability of these two models and two relationship matrices, the dataset was divided into the training and validation sets. Pigs born before September 7th, 2019 were considered as the training set, and a number of 150 pigs born after this time point were treated as the validation dataset. Prediction accuracy and dispersion were used to indicate the performance of different prediction models. Prediction accuracy was computed as the correlation between de-regressed proofs (DRP) and estimated breeding value (EBV), and dispersion was the deviation of regression coefficient (DRP on EBV) from 1. According to Garrick et al. (2009), two sets of DRP were calculated from EBV and reliabilities for animal model and RRM, respectively. Those EBV were computed from the phenotypes and pedigree information of all individuals. Reliabilities of EBV were obtained following the procedure proposed by Harris and Johnson (1998) for animal model, and by Jamrozik et al. (2000) for RRM. Solutions of RRM with both traditional and pedigree-and-genomic-based relationship matrices were used to calculate the average EBV over the test period.

## Results

In the analysis of RRM, considering the complexity of calculation of RFI in the statistical model, linear and second orders of the Legendre polynomials were tested (Table 2). The BIC values of RRM decreased as the order of polynomials for animal effect increased, while the order of polynomials for the other two random effects had no obvious tendency. Thus, a second-order Legendre polynomial was determined to be best for the additive effect, and a linear Legendre polynomial was fitted both for the permanent environmental effect and random litter effect.

**TABLE 2:** BIC trend for different orders of Legendre polynomials.

Parameters’ combinations	p	q	n	BIC
1	1	1	1	101,600.50
2	1	1	2	99,935.38
3	1	2	1	100,029.60
4	1	2	2	99,853.01
5	2	1	1	99,352.37
6	2	1	2	99,381.57
7	2	2	1	99,374.70
8	2	2	2	99,399.87

p is the pth-order Legendre polynomial for the additive effect, q is the qth-order Legendre polynomial for the permanent environmental effect, n is the nth-order Legendre polynomial for the litter random effect.

**FIGURE 2:**
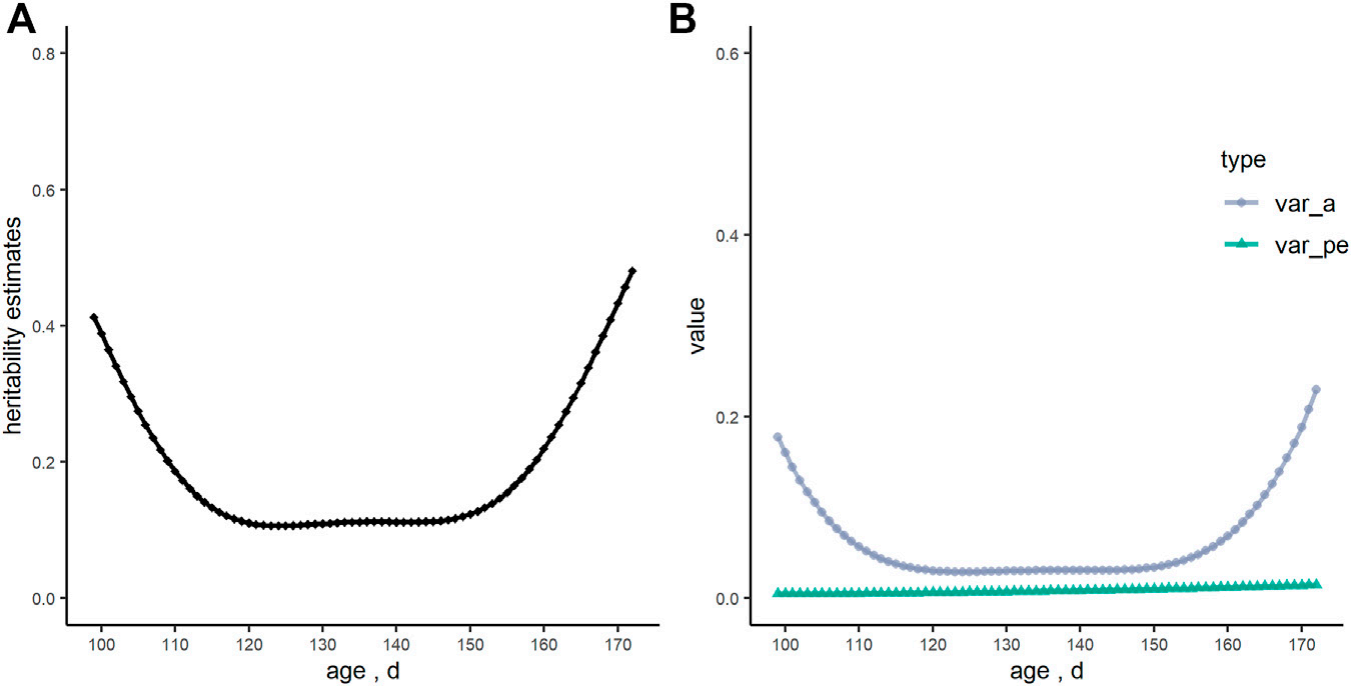
The tendency of heritability estimates (h^2^), genetic variance (var_a) and permanent environmental variance (var_pe) of residual feed intake (RFI, kg/d) over days in the random regression model. **(A)** heritability estimates; **(B)** genetic variance and permanent environmental variance.

The heritability estimate of RFI analyzed by the animal model for this Yorkshire boars’ population was 0.30. Figure 2 showed that heritability estimates of RFI using the RRM, and they were curves with dynamic changes with increasing age (ranged from 0.11 to 0.48). However, heritability estimates were quite stable from 120 to 150 days of age (ranged from 0.11 to 0.12). Meanwhile, the genetic variance of RFI had the same trend as heritability estimates, while the estimates of permanent environmental variance were constant.

The prediction accuracies and dispersion of these two models with two different relationship matrices were compared in Table 3. Compared to the animal model, accuracies for the RRM were increased by 24.2% (from 0.190 to 0.236) with **A** matrix and 40.9% (from 0.203 to 0.286) with **H** matrix. Prediction dispersion fluctuated among different models, and there was no obvious difference among these two models. Besides, the prediction accuracies and dispersion of the RRM-Hmat tended to be better than the RRM-Amat, and the combination of an RRM with **H** matrix ranked the highest prediction accuracy for all scenarios.

**TABLE 3:** The prediction accuracies and dispersion (|1-b|) of four kinds of models evaluating RFI.

Prediction models	Accuracies	Dispersion
Animal model-amat	0.190	0.187
Animal model-hmat	0.203	0.243
RRM-amat	0.236	0.233
RRM-hmat	0.286	0.190

Animal Model-Amat: the animal model with **A** matrix; Animal Model-Hmat: the animal model with **H** matrix; RRM-Amat: random regression model with **A** matrix; RRM-Hmat: random regression model with **H** matrix.

## Discussion

In this study, longitudinal data and cumulative data of feed intake for the whole test periods of a Yorkshire boars’ population were used, the prediction performance of the random regression model (RRM) based on pedigree and genomic information for RFI prediction was compared with a regular animal model. The RRM-Hmat could result in higher prediction accuracy, and the RFI has the potential to be used routinely in pig breeding to improve feed efficiency.

With regard to the genetic evaluation of RFI, four linear regression terms were added in the model: the average daily gain, adjusted back-fat thickness, the initial body weight at the start of the test, and the metabolic body weight. In previous studies, different factors were modeled for RFI prediction in terms of specific scenarios considered (Cai et al., 2008; Hoque et al., 2009; Fan et al., 2010). Different linear regression terms added in statistical models and data errors in measurement could partly influence the estimation of variance components and the heritability of RFI (Hoque et al., 2009). Therefore, it is necessary to test these effects in the model and estimate genetic parameters of different populations. The strategy of the Legendre polynomials for model optimization in the current analysis was mainly based on the convergence of variance component estimations as well as the BIC value, which was used to judge the quality of different models for large sample data (Vrieze, 2012). This strategy provided a feasible way of achieving an expected prediction performance, with computational efficiency. In the present study, the heritability of RFI in the RRM was higher both at the start and end of the test period, which demonstrated the different genetic backgrounds in pig growth. The same tendency of the heritability estimates during the whole process were also reported (Shirali et al., 2017b; Coyne et al., 2017; David et al., 2021). A permanent environmental effect was also included in our RRM models, and the estimates of permanent environmental variance changed gently, which indicated that RFI was less influenced by permanent environmental effects in our study.

The RRM was superior to the animal model in this study. The higher prediction accuracy was received in RRM than that of animal model, and RRM-Hmat had less prediction dispersion than RRM-Amat. Studies also showed a clear advantage on RRM in prediction in livestock species, such as mink (Shirali et al., 2015), chickens (Begli et al., 2016; Begli et al., 2018), and pigs (Coyne et al., 2017; Shirali et al., 2017a; Shirali et al., 2017b). An explanation is that, with the longitudinal model, the test period for each individual becomes more flexible and all data during the whole test period can be fully utilized. In addition, the estimates of heritability for longitudinal traits changes dynamically with time point measurements, reflecting the correlation between data of adjacent time points, which is suitable for dissecting the genetic background of longitudinal traits. Besides, a previous study showed that RFI had different genetic correlations with several feeding behavior traits at early or late stages, which also indicated the change in feed intake capacity of pigs (Shirali et al., 2017a).

The added genomic information could further increase the accuracy of genetic prediction. Several studies have shown the advantages of genetic evaluation with both pedigree and genotype data (Chen et al., 2011; Christensen et al., 2012; Su et al., 2012; Misztal et al., 2020). Kang et al. (2017) showed that the single-step random regression model had the highest accuracy and best unbiasedness, and achieved reliable prediction ability in the analysis of longitudinal traits. The superiority of genomic selection was also reflected in another study on chickens (Shirali et al., 2017b). However, more dispersion was observed in the animal model with **H** matrix than with **A** matrix. The possible reason could be the unsuitable parameters in **H** matrix for different kinds of models and data. Owing to the better effect in genomic selection by inching parameters in **H** matrix (Christensen et al., 2012), more parameter combinations need to be tried, and the most suitable combination of parameters for specific groups and traits may differ. Besides, the different dispersion between using **A** matrix and **H** matrix was not consistent for the animal model and RRM. This might be because small data sets could lead to overfitting in the complex models, such as RRM, and larger longitudinal data sets are likely needed to investigate this further.

Since the cost of feeding accounts for the largest proportion of the total production costs in the swine industry, the prediction accuracy of RFI is extremely important. The increased accuracy in RRM with H matrix could be helpful in pig breeding, and would contribute to select the breeding pigs with potential of higher feed efficiency.

In conclusion, by using an RRM, the heritability changes dynamically, which suggests different genetic variations throughout the test period. RRM can make the most use of longitudinal data, and RRM with H matrix obtained the highest prediction accuracy. Therefore, with the accumulation of longitudinal trait data such as RFI, RRM with H matrix can help in the genetic evaluation of pigs, and has the potential to contribute faster genetic improvement of feed efficiency. This kind of model can be applied to other longitudinal data as well.

## Data Availability

The raw data supporting the conclusions of this article will be made available by the corresponding authors, without undue reservation.
